# Long Non-Coding RNA 554 Promotes Cardiac Fibrosis via TGF-β1 Pathway in Mice Following Myocardial Infarction

**DOI:** 10.3389/fphar.2020.585680

**Published:** 2020-12-16

**Authors:** Bihui Luo, Zhiyu He, Shijun Huang, Jinping Wang, Dunzheng Han, Hao Xue, Peiying Liu, Xiaojun Zeng, Dongfeng Lu

**Affiliations:** ^1^ Department of Cardiovascular Medicine, The First Affiliated Hospital of Guangzhou Medical University, Guangdong, China; ^2^ Department of Cardiovascular Medicine, Yue Bei People’s Hospital, Guangdong, China

**Keywords:** cardiac fibrosis, lncRNA 554, TGF-β1, myocardial infarction, fibroblast

## Abstract

**Rationale: **Cardiac fibrosis is observed in nearly every form of myocardial disease. Long non-coding RNAs (lncRNAs) have been shown to play an important role in cardiac fibrosis, but the detailed molecular mechanism remains unknown.

**Object: **We aimed at characterizing lncRNA 554 expression in murine cardiac fibroblasts (CFs) after myocardial infarction (MI) to identify CF-enriched lncRNA and investigate its function and contribution to cardiac fibrosis and function.

**Methods and Results: **In this study, we identified lncRNA NONMMUT022554 (lncRNA 554) as a regulator of MI-induced cardiac fibrosis. We found that lncRNA 554 was significantly up-regulated in the mouse hearts following MI. Further study showed that lncRNA 554 was predominantly expressed in cardiac fibroblasts, indicating a potential role of lncRNA 554 in cardiac fibrosis. *In vitro* knockdown of lncRNA 554 by siRNA suppressed fibroblasts migration and expression of extracellular matrix (ECM); while overexpression of lncRNA 554 promoted expression of ECM genes. Consistently, lentivirus mediated *in vivo* knockdown of lncRNA 554 could inhibit cardiac fibrosis and improve cardiac function in mouse model of MI. More importantly, TGF-β1 inhibitor (TEW-7197) could reverse the pro-fibrotic function of lncRNA 554 in CFs. This suggests that the effects of lncRNA 554 on cardiac fibrosis is TGF-β1 dependent.

**Conclusion: **Collectively, our study illustrated the role of lncRNA 554 in cardiac fibrosis, suggested that lncRNA 554 might be a novel target for cardiac fibrosis.

## Introduction

Cardiovascular diseases are the leading cause of death in the world (Gourdie et al., 2016). Cardiac fibrosis is associated with nearly every form of myocardial diseases (Travers et al., 2016). Upon myocardial infarction (MI), cardiac fibroblasts (CFs) begin to activate and remodel myocardium by secreting excessive extracellular matrix (ECM), leading to the stiffness and reduced compliance of heart. Excessive ECM deposition is a main contributor in the progression of heart failure and other forms of cardiac disease (Kong et al., 2014; Travers et al., 2016). Numerous studies have shown that TGF-β1 pathway is the master pathway in cardiac fibrosis. TGF-β1 exerts its effects by binding to TGF-β receptors type I and II (TβRI and TβRII) and in turn activates Smad 2 and Smad 3. Activated Smad 2 and Smad 3 can bind to Smad 4, and these heterometric complexes then translocate to nucleus and function as transcription factors that trigger pro-fibrotic gene transcription (Ma et al., 2018; Frangogiannis, 2019). TGF-β1 is a central regulator of ECM deposition in injury-induced fibrosis in heart. TGF-β1-mediated ECM synthesis is essential for wound repair, but excess ECM deposition leads to fibrosis (Walton et al., 2017; Xie et al., 2019; Yin et al., 2019). Although the mechanism of cardiac fibrosis has been partly explored, the effective therapies that target fibrosis remain limited. Thus, a further characterization of the cellular and molecular mechanisms of cardiac fibrosis is needed to identify specific regulatory molecules and targets.

Non-coding RNAs, including miRNA, lncRNA, and circRNA, are reported to play a pivotal role in cardiovascular diseases (Liu et al., 2015; Tang et al., 2017; Sun et al., 2019). Recently, long non-coding RNAs (lncRNAs) have been regarded as regulators in cardiac fibrosis (Creemers and van Rooij, 2016; Sun et al., 2019; Zhuang et al., 2019). LncRNAs are defined as transcripts with more than 200 nucleotides in length and do not code protein (Xie et al., 2014; Lucas et al., 2018). The differential expression of lncRNAs has been shown to participate in cardiac pathologies. A current study indicated that lncRNA Meg3 was downregulated in a pressure overload murine model and inhibition of Meg3 could inhibit MMP-2 in murine hearts after cardiac stress, leading to reduced cardiac fibrosis and improved diastolic function (Piccoli et al., 2017). However, the understanding of the molecular mechanisms of lncRNAs in cardiac fibrosis remains unsatisfactory.

A research showed that lncRNA NONMMUT022554, which we named as lncRNA 554 in current study, was highly up-regulated in a mouse model of cardiac fibrosis induced by MI (Qu et al., 2016). In the other study, Jiang et al. reported that lncRNA 554 was up-regulated in pulmonary fibroblasts and promoted lung fibrosis by interacting with miR-26a (Jiang et al., 2018). However, the molecular mechanism of lncRNA 554 in the process of cardiac fibrosis remain largely unknown.

The role of TGF-β1/Smad3 signaling pathway in fibrosis has been clearly identified. Although Smad3 has been considered to be a key transcription factor activated in response to many fibrogenic mediators, knockout of Smad3 can cause autoimmune disease (Roberts et al., 2006). To date, no specific treatment is available to mitigate cardiac fibrosis. Therefore, alternative approaches to inhibit the TGF-β1/Smad3 pathway could be significant in suppressing fibrosis. Recently, some novel lncRNAs, such as Erbb4-IR, LRNA9884 and H19, have been found to be involved in TGF-β1/Smad3 signal pathway (Zhang et al., 1939; Sun et al., 2018; Wang et al., 2019). However, there is still limited understanding of how lncRNAs regulate the TGF-β1/Smad3 signaling pathway in cardiac fibrosis. Therefore, in the present work, we aim to study the potential role of lncRNA 554 on TGF-β1/Smad3 signaling pathway in cardiac fibrosis.

## Materials and Methods

### Animals

Male C57BL/6 mice (8–10 weeks old, 20–30 g) were purchased from the Experimental Animal Center of Guangzhou University of Chinese Medicine. Mice freely accessed food and water. All experimental procedures were reviewed and approved by the Experimental Animal Ethics Committee of Guangzhou Medical University.

### Isolation and Culture of Cardiac Fibroblasts and Cardiomyocytes From Neonatal Mice

Neonatal mice cardiac fibroblasts (CFs) and cardiomyocytes (CMs) were isolated as described previously (Liang et al., 2018). In brief, hearts from 1–3 days-old C57BL/6 mice were completely minced and placed together in 0.25% trypsin. Cell suspensions were centrifuged and then resuspended in Dulbecco’s modified Eagle’s medium (DMEM) supplemented with 10% fetal bovine serum (FBS), 100 U/mL penicillin and 100 μg/ml streptomycin. Cardiac fibroblasts and cardiomyocytes were isolated and cultured at 37°C with 5% CO2. Passage 2–3 CFs were used for experiment.

### Small Interfering RNA Transfection

siRNA probe against lncRNA 554 was designed and produced by RiboBio (Guangzhou, China). And the nucleotide sequences of lncRNA 554 siRNA were 5′-GCA​GAU​UCU​UGC​CCU​ACU​UTT-3′, 5′-AAG​UAG​GGC​AAG​AAU​CUG​CTT-3’. Before transfection, cardiac fibroblasts were seeded in six well plate at 1.5 × 10 ^^5^ cells/well concentration and then cultured 24 h. And the fusion degree of cells reached 50% when transfected with siRNA. The scrambled control siRNA (NC) and lncRNA 554 siRNA (si-Lnc554) (20 nM) were transfected into cardiac fibroblasts respectively by using RiboFECT CP Transfection Kit. 48 h after transfection, the cells were used for the following experiments.

### Lentivirus Transfection

The Lentivirus overexpression lncRNA 554 was synthesized by Genechem (Shanghai, China). Isolated CFs were seeded at 40% confluence. And the transduction was performed 72 h after seeding. For Lentivirus transfection, Lentivirus-lncRNA 554 (Len-Lnc 554) or Lentivirus-negative control (Len-NC) was added to the cells with a multiplicity of infection of 100 MOI. After 72 h of transfection, the cells were collected.

### Cell Scratch Assay

According to the best transfection conditions of siRNA, NC and si-Lnc554 were used to transfect cardiac fibroblasts for 48 h. The cells were trypsinized and seeded in six well plate at 5 × 10 ^^5^ cells/well concentration and then cultured 12 h. Subsequently, a 1 ml pipette tip was employed to draw 3 parallel lines in each hole. The cells were washed with PBS for three times and then serum free medium was added to each well. The medium should be changed every 2 h for 3 times. The cells were cultured at 37°C with 5% CO2. The images were taken in 0, 12, 24 h after culture.

### Fluorescence *in situ* Hybridization

Fluorescence *in situ* hybridization was performed as described previously (Guo et al., 2018). CFs were rinsed in 1 x PBS and then fixed in 4% paraformaldehyde for 10 min at room temperature. Cells were rinsed in 1 x PBS at 4°C. 200 ml of Pre-hybridization Buffer was added at 37°C for 30 min. Hybridization was carried out with a FISH probe (Ribo^TM^ lncRNA 554 FISH Probe Mix (Red)) at 37°C in the dark overnight using Ribo^TM^ Fluorescent *In Situ* Hybridization Kit (C10910, RiboBio). The cells were washed with Wash Buffer I, II, and III at 42°C in the dark. The cells were stained with DAPI in dark and then washed with 1 x PBS three times. All images were obtained with a fluorescence or confocal microscope (LSM880; Zeiss, Jena, Germany).

### RNA Isolation, cDNA Generation and Quantitative Real-Time PCR

Total RNA was extracted from neonatal mouse CFs or left ventricular infarct border zone of heart using Trizol reagent (Takara, Japan). For each sample, 1 μg of the total RNA was converted to cDNA according to the manufacture’s instructions using the cDNA Reverse Transcription Kit (Takara, Japan). RNA levels of lncRNA 554, collagen I (Col 1), collagen III (Col 3), CTGF and fibronectin 1 (Fn 1) were detected using SYBR Green method (Takara, Japan) on the ABI Stepone Plus Fast Real-time PCR system. After circle reaction, the threshold cycle (Ct) was determined and the relative quantitative expression of lncRNA 554 and ECM mRNAs was calculated using method 2^−ΔΔCt^ and normalized to GAPDH as an internal control. The sequences of primers were synthesized by Sangon Biotech Co. Ltd (Shanhai, China). The sequences of lncRNA 554 primers were forward: 5′-CAT​GGA​TGC​AGG​CAG​TGA​TT-3’; reverse: 5′- GCC​TAG​AGT​TGG​CTT​GCT​TCT​T-3’. The sequences of Col 1 primers were forward: 5′-CAA​TGG​CAC​GGC​TGT​GTG​CG-3’; reverse: 5′-CAC​TCG​CCC​TCC​CGT​CTT​T GG-3’. The sequences of Col 3 primers were forward: 5′-TGGCACAGCAGTC CAACGTA-3’; reverse: 5′-AAG​GAC​AGA​TCC​TGA​GTC​ACA​GAC​A-3’. The sequences of Fn 1 primers were forward: 5′-ATG​TGG​ACC​CCT​CCT​GAT​AGT-3’; reverse: 5′- GCC​CAG​TGA​TTT​CAG​CAA​AGG-3’. The sequences of GAPDH primers were forward: 5′- AGG​TCG​GTG​TGA​ACG​GAT​TTG-3’; reverse: 5′-GGGGTCGTT GATGGCAACA-3’. The sequences of CTGF primers were forward: 5′-CTCCTA CTA​CGA​GCT​GAA​CCA​G-3’; reverse: 5′-CCA​GAA​AGC​TCA​AAC​TTG​ACA​GGC-3’.

### Mouse Model of Myocardial Infarction and *in vivo* Infection of Lentivirus

Mice were anesthetized with pentobarbital (40 mg/kg, i. v.) and were randomly divided into a sham-operated group, an MI group, a lentivirus group (Len-sh-lnc554) and a lentivirus control (Len-scramble) group. Their chests were opened to expose the hearts. The left descending coronary artery (LAD) was ligated with a 7/0 nylon suture to induce myocardial infarction. Myocardial ischemia was confirmed by markedly S-T segment elevation with electrocardiographic measurements. The sham-operated mice underwent the same process as the MI group but without ligation of LAD. After MI surgery, the mice were injected with lentivirus. The procedure was performed as described previously (Wang et al., 2018). A total of 30 μL of lentivirus (1 × 10^^9^ TU/mL for each mouse) including lentivirus vector and lentivirus-negative control (Genechem, Shanghai, China) were injected near the ligation line at four sites. The shRNA sequences of lncRNA 554 were: 5′-GCA​GAT​TCT​TGC​CCT​ACT​T-3′, 5′-AAGTAGGGC AAGAATCTG-3’. The sequences were inserted into GV248 lentiviral vector (Genechem). At 0, 3, 7, 14, and 28 days after the operation, the mice in the MI group were sacrificed to detect the expression of lncRNA 554, and the remaining mice were fed and monitored daily until the second week for further study.

### Masson Trichrome Staining and Hematoxylin and Eosin Staining

Masson's trichrome staining and hematoxylin and eosin (H&E) staining were performed to estimate collagen deposition in the hearts. Two weeks after MI surgery, the hearts were dissected out and fixed with 4% paraformaldehyde, embedded in paraffin, and cut into a 5 µm thick cross section. The sections were stained with Masson's trichrome staining kit (Sigma-Aldrich LLC, United States) and hematoxylin and eosin kit (Solarbio, Beijing), and collagen deposition was examined using Ortho Microscope (Nikon, Japan). The area of fibrosis in each group was calculated by ImageJ software.

### Echocardiography

Transthoracic echocardiography was performed at 14 days after MI surgery using a Vevo®2,100 High-Resolution Imaging system (Visual Sonics) to monitor changes in left ventricular function. The mice were anesthetized with isoflurane, two-dimensional ultrasound and M-mode ultrasound measurements were performed. The short axis at the level of the parasternal papillary muscle of each mouse was measured. And some parameters such as left ventricular fractional shortening (LVFS), left ventricular anterior wall end diastolic thickness (LVAWd), left ventricular anterior wall end systolic thickness (LVAWs), left ventricular internal diastolic diameter (LVIDd), left ventricular internal systolic diameter (LVIDs), and left ventricular ejection fraction (LVEF) were measured.

### Statistical Analysis

The experimental data were analyzed by SPSS 16.0 and GraphPad Prism5.0 software, and the results were expressed as mean ± standard deviation (x ± s). The comparison between the two groups was analyzed by *t*-test, and one-way AVOVA was used for multiple comparisons. The results were considered statistically significant when *p* < 0.05.

## Results

### LncRNA 554 Is Upregulated After Myocardial Infarction and Is Enriched in Cardiac Fibroblasts

**FIGURE 1:**
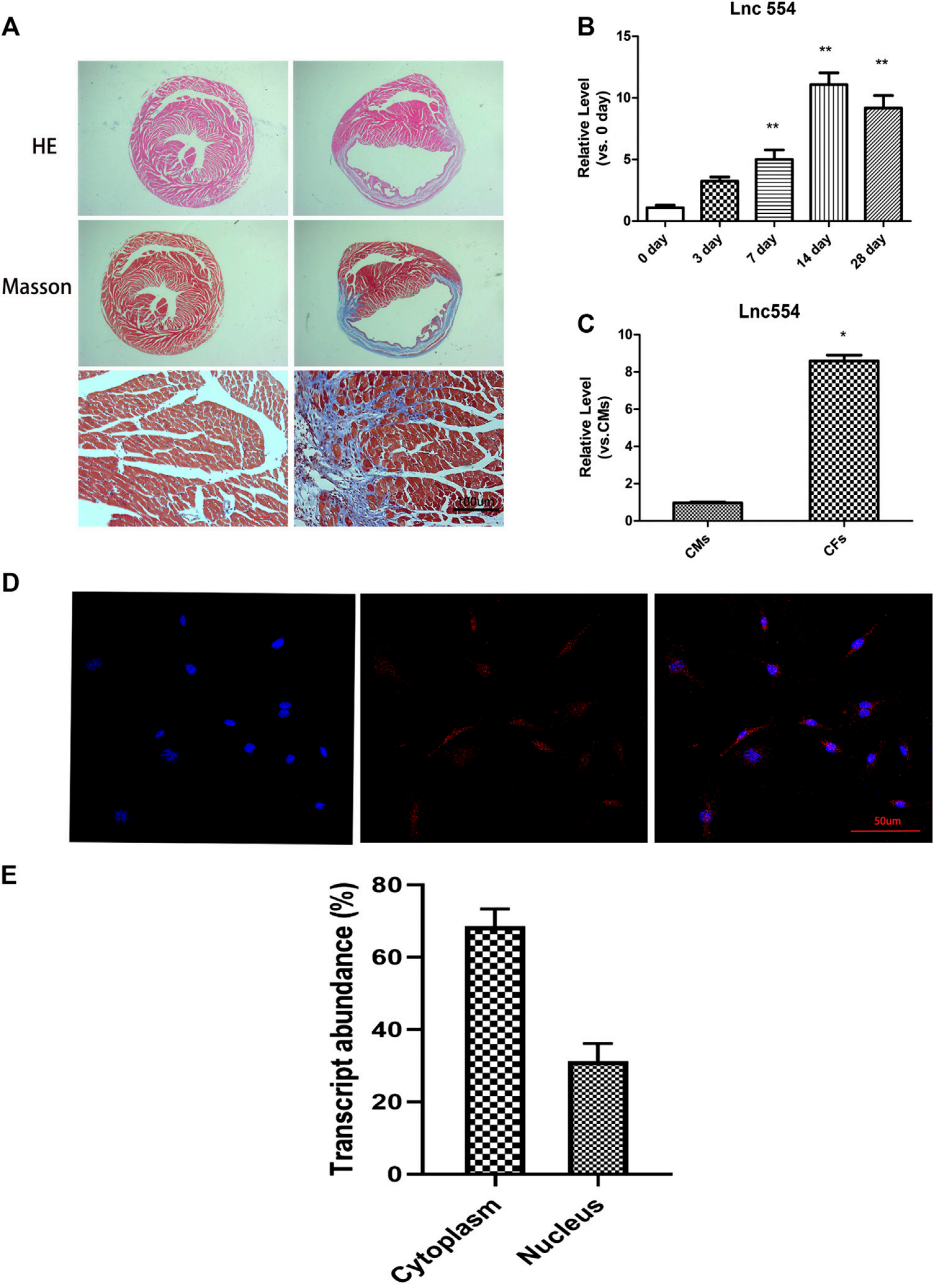
LncRNA 554 is upregulated after myocardial infarction and is enriched in cardiac fibroblasts. Two weeks after MI, the hearts of mice were stained by hematoxylin-eosin staining and Masson’s trichrome staining **(A)**. Scale bars, 100 μm n = 8 mice in each group; **(B)** qRT-PCR were conducted to detect the expression of lncRNA 554 in the border zone of mice at different time (0, 3, 7, 14, 28 days) after MI. **p < 0.01 vs 0 day, n = 8; **(C)** qRT-PCR were conducted to detect lncRNA 554 levels in CMs and CFs. *p < 0.05 vs CMs, n = 5. Representative fluorescent *in situ* hybridization images; **(D)** and corresponding statistical chart; **(E)** showing the expression abundance and cellular localization of lncRNA 554 in CFs. Scale bars, 50 μm n = 10.

To determine the potential role of lncRNA 554 in cardiac injury and repair, mouse MI model was used. Fourteen days after MI, HE staining and Masson’s trichrome staining were used to determine cardiac fibrosis. The ventricular wall became thinner and the interstitial fibrotic area was significantly increased in MI hearts as compared with that in SHAM control hearts (Figure 1A). As shown in Figure 1B, the level of lncRNA 554 in border zone was elevated after MI and peaked on 14 days post-MI. To determine the distribution of lncRNA 554 in heart, qRT-PCR was conducted in fibroblasts and cardiomyocytes respectively. Our results showed that lncRNA 554 was enriched in CFs compared to cardiomyocytes (CMs) (Figure 1C), suggesting lncRNA 554 might be an important regulator in cardiac fibrosis. Next, we detected the subcellular distribution of lncRNA 554 in CFs by using fluorescence *in situ* hybridization to label lncRNA 554. The results showed that lncRNA 554 was expressed in both cytoplasm and nucleus of cardiac fibroblasts (Figures 1D,E).

### Knockdown of LncRNA 554 Attenuates the Function of Cardiac Fibroblasts and Reduces the Expression of TGF-β1 and Its Downstream

**FIGURE 2:**
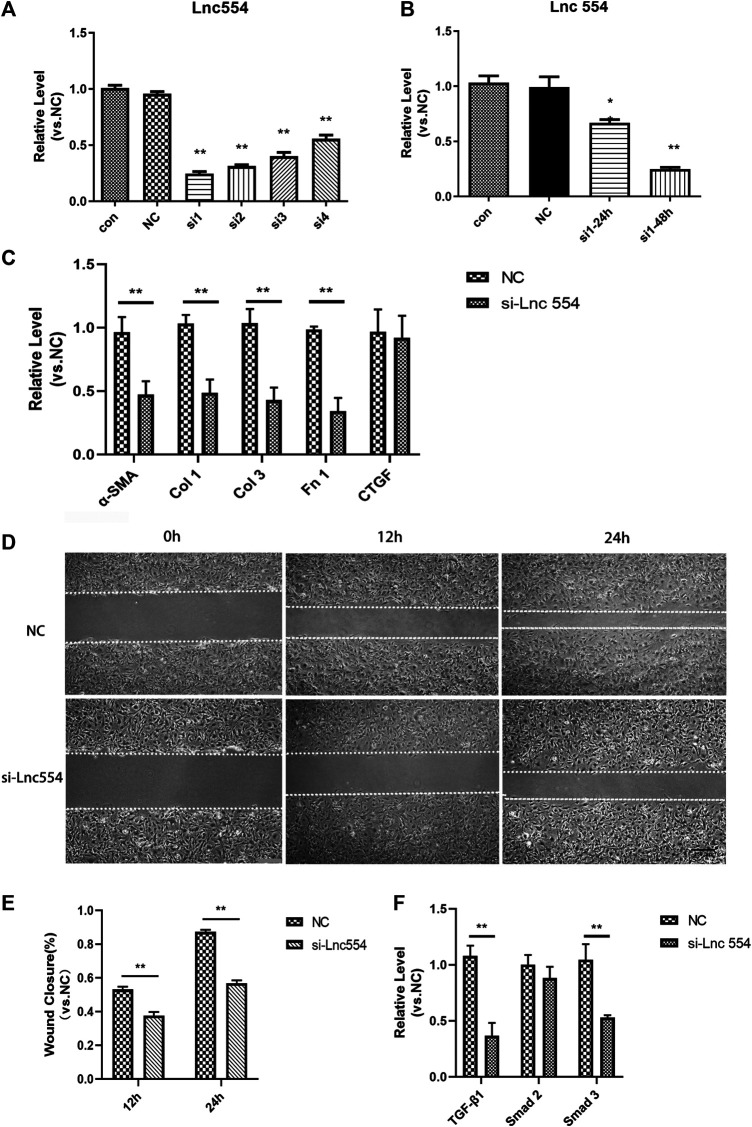
Knockdown of lncRNA 554 attenuates the function of cardiac fibroblasts and reduces the expression of TGF-β1 and its downstream **(A)** qRT-PCR was used to detect the expression of lncRNA 554 in CFs after siRNA transfection. Control, untreated group; NC, scrambled control siRNA transfection group; Si1-4, siRNA transfection group. **p < 0.01 vs NC, n = 5; **(B)** The CFs was transfected with si1 with different time (24 h, 48 h), and then qRT-PCR was conducted to detect the level of lncRNA 554. **p < 0.01 vs NC, n = 5; **(C)** After silencing of lncRNA 554, the level of ECM mRNAs (α-SMA, Col 1, Col 3, Fn 1, CTGF) were analyzed by qRT-PCR. **p < 0.01 vs NC, n = 5; **(D)** and **(E)** The efficiency of healing in lncRNA 554 silencing group and NC group was analyzed by wounding healing assay at different time (0, 12 and 24 h). **p < 0.01 vs NC, n = 5. Scale bars, 250 μm; **(F)** After silencing of lncRNA 554, qRT-PCR was conducted to detect the level of TGF-β1 pathway (TGF-β1, Smad 2, Smad 3). **p < 0.01 vs NC, n = 5.

To explore the functions of lncRNA 554 in cardiac fibroblasts, we used siRNA to down-regulate the expression of lncRNA 554. We screened four potential siRNAs sequences, and found that the first siRNA (Si1) had the highest silencing efficiency (Figure 2A), and could significantly down-regulate the expression of lncRNA 554 48 h after transfection (Figure 2B). Knockdown of lncRNA 554 (si-Lnc554) could remarkably reduce α-SMA, collagen and fibronectin production, but had no effect on connective tissue growth factor (CTGF). In contrast, control siRNA, NC, did not show influence on collagen production (Figure 2C). This suggests that lncRNA 554 may promote the activation of myofibroblasts and the synthesis of extracellular matrix to induce cardiac fibrosis. Upon injury, cardiac fibroblasts are activated and migrate to the injury area to repair. We used cell scratch assay to identify the migration of CFs, and our findings showed that silencing of lncRNA 554 could significantly inhibit CFs migration as compared to control group (Figures 2D,E). As it is well known, TGF-β1 pathway plays an important role in fibrotic remodeling by promoting synthesis and secretion of ECM proteins in cardiac fibroblasts. Since we have found that lncRNA 554 was capable to regulate the process of fibrosis and was enriched in fibroblasts, we next tested whether lncRNA 554 could regulate cardiac fibrosis via TGF-β1 pathway. We found that knockdown of lncRNA 554 could significantly down-regulate the expression of TGF-β1 and Smad 3, but had no significant effect on Smad 2 (Figure 2F). This suggests that lncRNA 554 may play a role in promoting fibrosis through TGF-β1 signal pathway.

### LncRNA 554 Regulates Cardiac Fibrosis *via* TGF-β1 Signal Pathway

**FIGURE 3:**
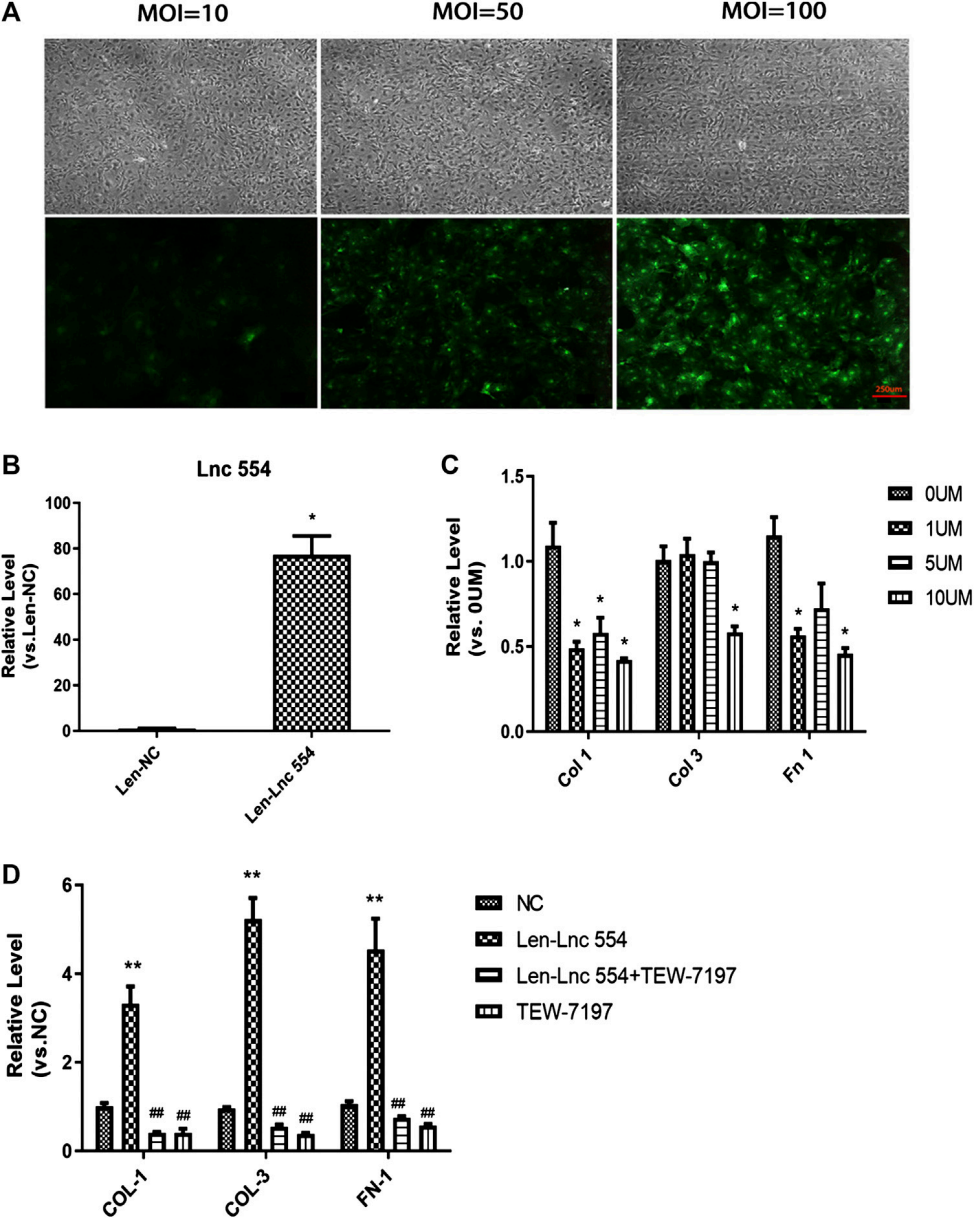
LncRNA 554 regulates cardiac fibrosis via TGF-β1 signal pathway *in vitro* **(A)** Representative images of GFP fluorescence intensity of different MOI (10, 50 and 100) show the infection efficiency of lentivirus. n = 5. Scale bars, 250 μm; **(B)** The level of lncRNA 554 overexpression by lentivirus was analyzed by qRT-PCR. *p < 0.05 vs NC, n = 5; **(C)** The level of ECM mRNA in CFs treated with different concentration TGF-β1 inhibitor (TEW-7197) (0 , 1, 5 and 10 μM). *p < 0.05 vs 0 μM, n = 5; **(D)** The CFs were treated with lentivirus-NC, lentivirus-lncRNA554 and (or) TEW-7197. qRT-PCR was conducted to detect ECM levels (Col 1, Col 3, Fn 1). **p < 0.01 vs NC, ##*p* < 0.01 vs Len-Lnc 554, n = 5. NC or Len-NC, empty lentivirus group; Len-Lnc554, lncRNA 554 overexpression group.

As knockdown of lncRNA 554 led to reduction of collagen, we tested whether upregulation of lncRNA554 could increase the expression of collagen-related genes. First of all, in order to screen the best infection conditions, we set up three groups of lentivirus with different concentrations (MOI = 10, 50, 100) to infect fibroblasts. The results showed that the efficiency of lentivirus infection was the highest when MOI = 100 was used (Figure 3A). Next, we detected the expression of lncRNA 554 in MOI = 100, and the results showed that the expression of lncRNA 554 in overexpression group was significantly higher than that in empty virus group (Figure 3B). And we found that the expression of collagen I, collagen III and fibronectin was significantly increased after overexpression of lncRNA 554 (Figure 3D). Next, to further verify that lncRNA 554 may be involved in cardiac fibrosis through the TGF-β1 signal pathway, we applied TGF-β1 inhibitor (TEW-7197) to treat fibroblasts with overexpression of lncRNA 554. Firstly, we set the concentration according to the study of Zhao et al., to determine the best concentrations of TEW-7197 to inhibit the effect of TGF-β1 on fibrosis (Zhao et al., 2018). And we found that 10 μM TEW-7197 administration had the best efficiency to inhibit fibrotic factors (Figure 3C), so this concentration was applied in the following experiments. Interestingly, TGF-β1 inhibitor (TEW-7197) treatment significantly reversed the fibrosis induced by lncRNA554 overexpression (Figure 3D). Based on these, we believed that lncRNA 554 regulated the process of fibrosis, at least partially, through TGF-β1 pathway.

### Knockdown of LncRNA 554 Mitigates Cardiac Fibrosis of Infarcted Hearts

**FIGURE 4:**
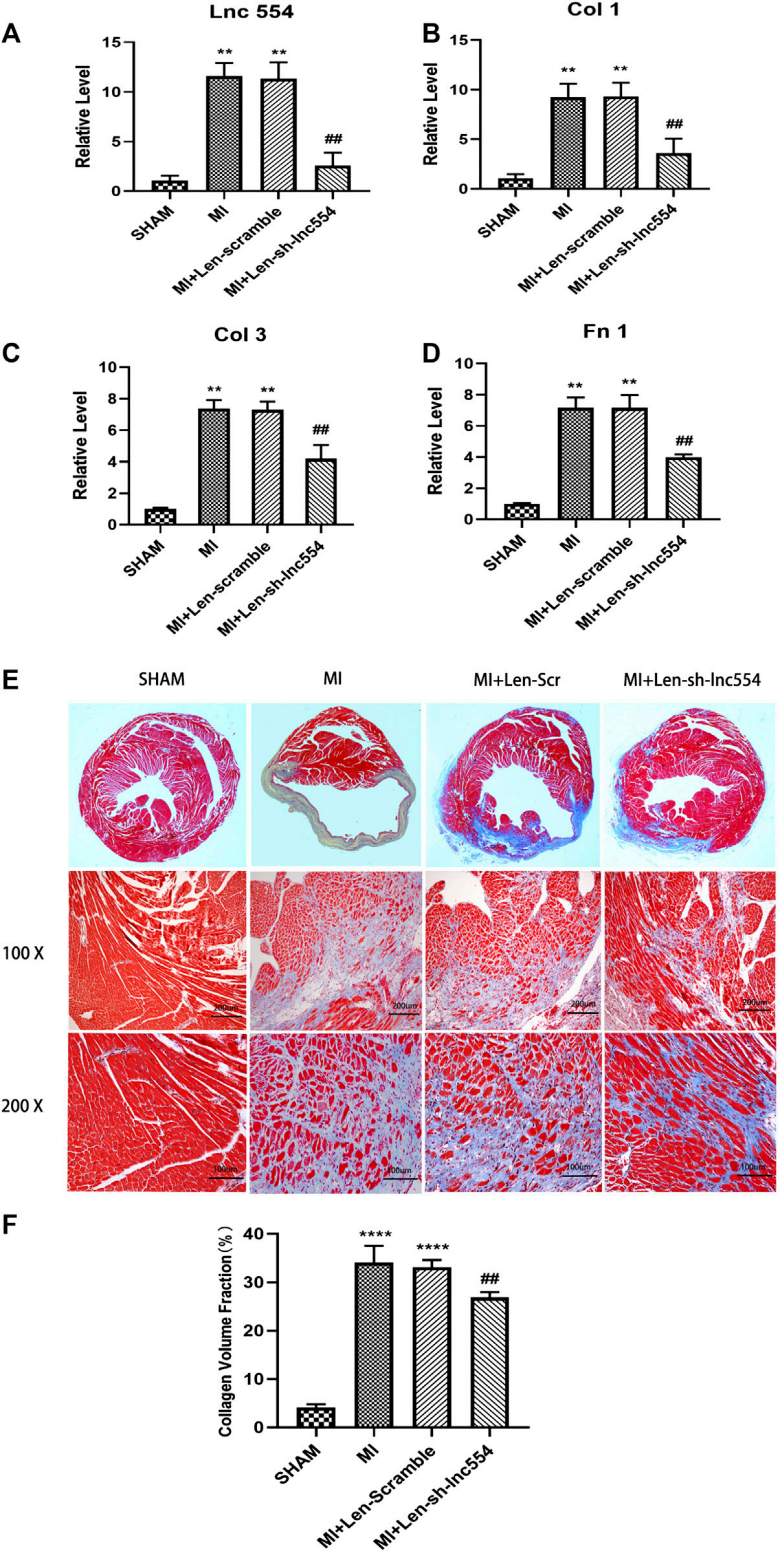
Knockdown of lncRNA 554 mitigates cardiac fibrosis of infarcted hearts. To knockdown lncRNA 554, the hearts were injected with lentivirus containing control or lncRNA 554 shRNA and subjected to qRT-PCR and Masson’s trichrome staining in 14 days after MI **(A)** The expression of lncRNA 554 was detected by qRT-PCR in post-MI hearts. **p < 0.01 vs SHAM, ##*p* < 0.01 vs MI + Len-scramble, n = 8. The expression of Col 1; **(B)**, Col 3; **(C)** and; Fn 1 **(D)** mRNA in peri-infarct area. **p < 0.01 vs SHAM, ##*p* < 0.01 vs MI + Len-scramble, n = 8; **(E)** and **(F)** Representative images of Masson’s trichrome staining and a quantification of Masson’s trichrome staining show the degree of fibrosis. ****p < 0.0001 vs SHAM, ##*p* < 0.01 vs MI + Len-scramble, n = 8.

Having proved that down-regulation of lncRNA 554 significantly reduced the expression of fibrosis-related genes *in vitro*, we went ahead to explore what would happen following lncRNA 554 silencing *in vivo*. The hearts were injected with lentivirus containing control or lncRNA 554 shRNA after MI. As shown in Figure 4A, the expression of lncRNA 554 was remarkably down-regulated. The main proteins in ECM, collagen I, collagen III and Fn 1, were largely decreased at mRNA levels by lncRNA 554 knockdown, whereas control shRNA had no such effect (Figures 4B–D). To estimate the degree of fibrosis after MI surgery, the hearts were stained with Masson’s trichrome staining. We found that lncRNA 554 down-regulation markedly reduced the blue staining, which represents reduced fibrotic region as compared to that in control group (Figures 4E,F). Taken together, our study suggests that lncRNA 554 plays an important role in MI-induced cardiac fibrosis.

### Knockdown of LncRNA 554 Improves Cardiac Function of Infarcted Hearts

**FIGURE 5:**
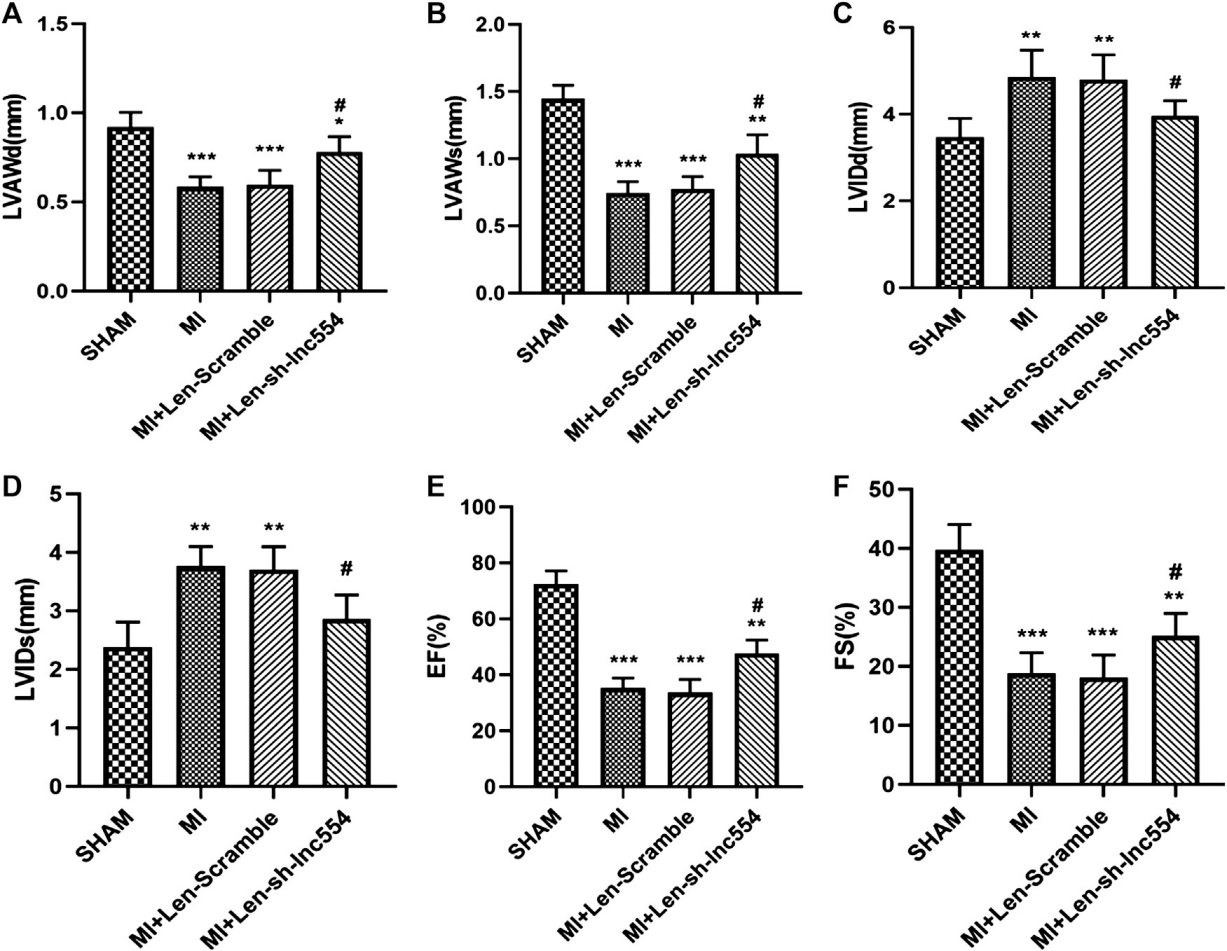
Knockdown of lncRNA 554 improves cardiac function of infarcted hearts. The hearts were injected with lentivirus containing control or lncRNA 554 shRNA after MI. Two weeks after MI, ventricular parameters were measured and analyzed by echocardiography, including LVAWd; **(A)**, LVAWs; **(B)**, LVIDd; **(C)**, LVIDs; **(D)**, left ventricular EF; **(E)**, and FS; **(F)**. Data are presented as mean ± SEM. *p < 0.05, **p < 0.01, ***p < 0.001 vs SHAM group; #*p* < 0.05, ##*p* < 0.01 vs MI + Len-Scramble group, n = 8. EF: ejection fraction; FS: fractional shortening; LVAWd: left ventricular anterior wall end diastolic thickness; LVAWs: left ventricular anterior wall end systolic thickness; LVIDd: left ventricular internal diastolic diameter; LVIDs: left ventricular internal systolic diameter.

To identify whether knockdown of lncRNA 554 influences cardiac function of infarcted hearts, echocardiography was performed. MI greatly compromised the function of hearts, reflected by decreases in LVAWd (Figure 5A) and LVAWs (Figure 5B) and increases in LVIDd (Figure 5C) and LVIDs (Figure 5D). Consistent with above deteriorations, MI dramatically reduced EF (Figure 5E) and FS (Figure 5F). Inhibiting lncRNA 554 by lentivirus mitigated MI-induced deleterious changes (Figures 5A–D) and improved cardiac functions (Figures 5E,F). These data suggest that lncRNA 554 plays a critical role in MI-induced structural remodeling and dysfunction, which is due to its capacity of regulating fibrosis process.

## Discussion

In the present study, we revealed a novel function and molecular mechanism of lncRNA 554 in MI-induced cardiac fibrosis. We first observed upregulation of lncRNA 554 on day 3 post MI, and it reached peak on day 14 and stayed high till day 28 post MI. Furthermore, we found that expression of lncRNA 554 was highly enriched in CFs compared to that in cardiomyocytes. Consistently, down-regulation of lncRNA 554 remarkably reduced CFs migration and ECM genes level *in vitro* and we found that silence of lncRNA 554 mitigated cardiac fibrosis and protected cardiac functions of MI mice. More importantly, TGF-β1 inhibitor could evidently inhibit the function of lncRNA 554, indicating that pro-fibrotic function of lncRNA 554 was at least partly owing to TGF-β1 signal pathway.

LncRNAs have recently been reported to regulate a variety of pathophysiological processes of cardiac diseases, including cardiac fibrosis (Wang et al., 2015; Jiang and Zhang, 2017; Greco et al., 2018). A recent study from Micheletti et al. has shown that lncRNA Wisper (Wisp2 super-enhancer–associated RNA) in CFs regulates cardiac fibrosis after myocardial infarction (Micheletti et al., 2017). In another study, lncRNA H19 was significantly upregulated in the infarct area in mouse model and acted to antagonize Y-box-binding protein (YB)-1 through direct interaction, resulting in cardiac fibrosis (Choong et al., 2019). Another study showed that knockdown of lncRNA MIAT reduced cardiac fibrogenesis by increasing the expression of miR-24, which targeted Furin, a component of the TGF-β1 signal pathway (Qu et al., 2017). Our findings showed that overexpression lncRNA 554 could increase ECM genes level and TGF-β1 inhibitor could reverse this function. In other word, lncRNA 554 promoted cardiac fibrosis at least partly via TGF-β1 signal pathway.

As our FISH experiment found that the subcellular localization of lncRNA 554 was in both cytosol and nucleus, our future studies will focus on determining the potential cytosolic and nuclear factors of TGF-β1 pathway that can be regulated by lncRNA 554. Recently, lncRNAs have been reported to act as miRNA sponges in regulating the biological activities of cardiac fibroblasts (Uchida and Dimmeler, 2015; Huang, 2018). Liang et al. found that lncRNA PFL promoted fibroblast-myofibroblast transition by competitively binding let-7 days, which inhibited the expression of platelet-activating factor receptor (PTAFR) (Liang et al., 2018). In addition, a previous study illustrated that lncRNA MIAT could absorb miR-24 through its sponge-like action, leading to down-regulation of miR-24 and contribute to cardiac fibrosis (Qu et al., 2017). Moreover, Jiang et al. reported that lncRNA 554 was up-regulated in pulmonary fibroblasts and promoted lung fibrosis by interacting with miR-26a (Jiang et al., 2018). Thus, we hypothesize that lncRNA 554 may act as sponge of miRNA, and this results in cardiac fibrosis. In our future work, we will investigate the effect of lncRNA 554 on the regulation of miRNA during cardiac remodeling.

However, there are still some limitations of the present study. Firstly, the detailed mechanism of myocardial fibrosis mediated by lncRNA 554 through TGF-β1 signaling pathway, especially which downstream target genes are directly or indirectly affected, needs to be further validated in future studies. In addition, we need to detect the level of lncRNA 554 in patients with MI in the future, and evaluate its clinical value. Besides, since there are many mechanisms and factors leading to cardiac fibrosis, whether lncRNA 554 promotes cardiac fibrosis through other mechanisms, such as inflammation, still needs to be further verified.

In summary, our results revealed lncRNA 554 as a critical pro-fibrotic lncRNA that promoted collagen synthesis, and myocardial fibrosis, and the mechanism may involve the activation of TGF-β1 signal pathway. Knockdown of lncRNA 554 represents a promising strategy for the intervention of cardiac fibrosis.

## **Data Availability Statement**

The raw data supporting the conclusions of this article will be made available by the authors, without undue reservation, to any qualified researcher.

## **Ethics Statement**

The animal study was reviewed and approved by Experimental Animal Ethics Committee of Guangzhou Medical University.

## Author Contributions

ZH, SH, JW and DH performed the research, HX and PL analyzed the data, BL, XZ, JW, and DH wrote the paper, BL and DL designed the research study. All authors contributed to manuscript revision, read, and approved the submitted version.

## Funding

This study was supported by National Natural Science Foundation of China (81570258), Natural Science Foundation of Guangdong Province of China (2018A030313060), Medical Science and Technology Research Foundation of Guangdong Province of China (A2018191, A2020178, A2020284), Guangzhou Health Science and Technology Project (20201A011072) and College Students' Science and Technology Innovation Project (2019A002).

## Conflict of Interest

The authors declare that the research was conducted in the absence of any commercial or financial relationships that could be construed as a potential conflict of interest.
